# Personalized Steering Feel Control Based on Driving Style Recognition and Closed-Loop Motion Regulation

**DOI:** 10.3390/s25247686

**Published:** 2025-12-18

**Authors:** Hsin Guan, Yimeng Song, Pingping Lu, Chao Dai, Shenzhen Gao, Jun Zhan, Chunguang Duan, Yinsheng Liao, Binggen Zhao

**Affiliations:** 1 The National Key Laboratory of Automotive Chassis Integration and Bionics, Jilin University, Changchun 130022, Chinasongym23@mails.jlu.edu.cn (Y.S.); daichao22@mails.jlu.edu.cn (C.D.); gaosz24@mails.jlu.edu.cn (S.G.); anhduan@jlu.edu.cn (C.D.); 2 BYD Automotive Industry Company Ltd., Shenzhen 518118, China; liao.yinsheng@byd.com (Y.L.); zhao.binggen@byd.com (B.Z.)

**Keywords:** steering feel, driving style, motion control

## Abstract

Against the backdrop of the continuous expansion of the automotive industry, consumer demand is undergoing a profound shift from quantity to quality. Conventional steering systems, due to their lack of dynamic adaptation to driver styles, struggle to meet the diverse needs of different user groups. To address this challenge, this study proposes a personalized steering feel control strategy, which achieves precise control of target steering feel by integrating driving style identification and closed-loop control strategies. Subsequently, this study validates the effectiveness and robustness of the proposed control method using a driving simulator. The results demonstrate that the proposed control strategy delivers effective performance across multiple operating conditions, providing drivers with a comfortable and satisfying steering feel, thereby enhancing their driving experience.

## 1. Introduction

In recent years, with the rapid development of the automotive industry, consumers’ demands for vehicle-driving experience have been constantly increasing, with particular attention paid to the steering performance during driving. Steering feel refers to the vehicle’s steering characteristics perceived by the driver through the steering wheel, including key information such as torque feedback. Research on steering feel control methods can be traced back to the early development stage of electric power steering systems. In 1991, Shimizu, Y. et al. [1] first systematically expounded the basic control strategies of electric power steering, pointing out that it mainly includes assist control, return control, and damping control, laying the theoretical framework for subsequent research in this field. Additionally, they proposed an innovative stability analysis method and controller design concept for EPS systems. Lee, D. et al. [2] further pointed out that steering feel is mainly determined by the assist torque map, which reflects the mapping relationship between the driver’s perceived torque and the motor assist torque that varies with vehicle speed. However, due to the high gain and nonlinear characteristics of the system, relying solely on the torque map design is prone to cause system instability, manifested as steering oscillation or divergence. Therefore, in the control of electric power steering systems, it is necessary to simultaneously consider the design of the assist torque map and the comprehensive optimization of stability compensators. The primary role of the compensator is to enhance system robustness and effectively suppress oscillations. This ensures the smoothness and controllability of the steering process.

Fu et al. [3] proposed a stability controller design method based on dynamic characteristic analysis to address the torque oscillation and stability degradation problems caused by high assist gain and time-varying assist gain characteristics in the EPS system. By reconstructing the transfer function model from steering torque to sensor torque, the nonlinear impact of variable assist gain on system dynamic response was quantitatively evaluated. On this basis, a multi-objective optimization function centered on phase margin, gain margin, and crossover frequency was constructed. Subsequently, the optimal controller parameters were determined by maximizing the comprehensive stability index. This process significantly enhanced the system’s robustness. Nguyen et al. [4] proposed a Backstepping PID (BSPID)-integrated algorithm combining backstepping and PID control to improve the overall control performance of the EPS system. The designed BSPID cascade controller utilized an iterative optimization algorithm to adjust the gain coefficients of the control signal, thereby enhancing the system’s dynamic response capability. At the same time, a steering system dynamics model with five state variables was established, fully considering the influence of road reaction torque and external disturbances, improving the model’s completeness and practicality. Na et al. [5] proposed an improved active disturbance rejection control (ADRC) method to address the torque fluctuation problem caused by low-frequency disturbances (such as road resistance, mechanical friction, and motor parameter drift) in the EPS system. This method achieves online estimation and dynamic compensation of internal and external disturbances by constructing a target torque algorithm related to the steering wheel angle and angular velocity. By integrating this with the closed-loop control architecture of an enhanced ADRC, the approach further improves control accuracy and system stability.

Collier-Holman [6] first proposed an EPS control method based on torque closed-loop. This method compares the actual applied torque with the expected torque and uses the error signal to adjust the assist torque provided by the EPS system, effectively reducing the deviation between the actual steering torque and the target value, and achieving precise feedback control of the steering process. Lee et al. [7] developed an EPS torque feedback control algorithm to enhance the naturalness of the steering feel and the robustness of the system. This algorithm consists of a target torque generator and a target torque tracking controller, achieving the decoupling of steering feel and stability control functions. Among them, the target torque generator determines the ideal driver-perceived torque based on the estimated road reaction torque. Subsequently, Williams [8] further proposed a modular EPS target steering torque feedback controller, which can dynamically generate target torques suitable for different conditions based on the real-time rack force and achieve high-precision tracking through an independent steering torque feedback control module. Jose [9] (USA) introduced a reference torque model that integrates the influence of steering angle and angular velocity, and by adjusting the model parameters, the steering feel can be made adjustable, enhancing the adaptability of human–machine interaction. Nguyen [10] adopted the fuzzy linear quadratic tracking (FLQT) method, combining fuzzy logic with linear quadratic tracking (LQT). This FLQT algorithm takes angle error and angular velocity error as inputs and online corrects the control input of LQT, significantly reducing the system tracking error and ensuring the dynamic consistency and control accuracy of all state variables. Yang [11] proposed an EPS control framework for ground vehicles based on admittance control, selecting the steering wheel angle as the reference signal and using a PID controller to precisely track this signal, thereby shaping the ideal steering response characteristics. Wilhelm et al. [12] proposed an active friction compensation strategy for the column EPS system to fully consider the dynamic characteristics of friction. This control architecture adopts a dual-loop structure: the inner loop estimates the internal friction of the system in real time and performs feedforward compensation through motor input; the outer loop includes a frictionless reference model (acting as a trajectory planner) and a linear feedback controller. This structure minimizes the error between the actual system output and the reference model response, using steering wheel angle and angular velocity as tracking targets. Ultimately, this approach improves both control accuracy and the consistency of the steering feel. Li et al. [13] proposed a torque superposition control algorithm to optimize the steering feel, which consists of a reference model, a rack force estimator, and a tracking controller, achieving the reconstruction and approximation of the ideal steering characteristics. Kim [14] proposed a robust nonlinear torque control method based on the steering wheel torque model, directly feeding back the steering wheel torque as the core state variable. By tracking the reference torque determined by the steering angle, angular velocity, and vehicle speed through a nonlinear sliding mode control law, the dynamic response capability and anti-interference performance of the system were enhanced. Nguyen [15] proposed a composite control strategy integrating backstepping control, PID, and fuzzy logic. Among them, the fuzzy logic module dynamically adjusts the parameters of the PID controller, and its output serves as the input of the backstepping controller, forming a cascaded anti-disturbance structure, achieving near-zero error tracking of the steering motor angle. Li et al. [16] proposed a torque superposition steering feel control algorithm based on a feedback mechanism, which also includes a reference model, a rack force estimator, and a tracking controller. The reference model combines the rack force estimation results based on Kalman filtering to generate a reference steering feel that reflects the actual vehicle operating state. The sliding mode tracking controller applies superimposed torque to drive the actual system state to approach the output of the reference model, ensuring good dynamic tracking performance. Choi et al. [17] proposed a speed control type steering feedback method based on a admittance model, where the steering feel is generated by the admittance model. This model calculates the expected steering wheel angular velocity based on the driver’s input torque, and the steering feedback control module tracks this velocity command to achieve the expected steering feel. Shi et al. [18] developed a high-fidelity steering feel control strategy based on a terminal sliding mode rack force observer. Feedback characteristic curves for rack force and hand force compensation torque are designed according to different vehicle motion states to restore the real road feedback sensation. At the same time, active disturbance rejection control (ADRC) is used to regulate the voltage of the steering wheel motor, further enhancing the robustness and control accuracy of the system.

Existing steering feel control methodologies predominantly encompass two primary domains. The first involves the compensation of disturbances within the steering system, typically implemented via open-loop strategies. The second focuses on the modulation of assist characteristics through the establishment of torque- or angle-based closed-loop control architectures. The overarching technical paradigm can be delineated as follows: reference models generate target torque or angular signals, which are subsequently tracked with precision using closed-loop algorithms—such as proportional–integral–derivative (PID) control or model predictive control (MPC)—to regulate the assist output effectively. This framework facilitates mode switching among diverse steering feel profiles by dynamically tuning the parameters of the reference signals. However, a salient limitation of prevailing closed-loop approaches lies in their reference signal generation mechanisms. Most existing studies focus heavily on disturbance rejection or stability augmentation but overlook the dynamic modeling of drivers’ subjective perceptual preferences. Unlike traditional methods that rely on static assist maps or fixed reference models, which fail to adapt to individual driver variances, our proposed strategy integrates a driving style recognition module directly into the control loop. This allows for the real-time modulation of the reference model based on the identified ‘aggressive’, ‘normal’, or ‘conservative’ style, a feature largely absent in prior arts. Consequently, the system transforms from a passive assist mechanism to an active, personalized motion regulator.

## 2. Methodology of Driving Style Recognition

To achieve high-precision recognition of driving styles for matching steering feel expectations across different driver types, this study conducted the following work.

### 2.1. Regarding Data Acquisition

Data were collected via simulator-based experiments. Initially, a bespoke test scenario tailored to the data acquisition requisites was developed using RoadRunner R2024a software. This scenario incorporated eleven distinct curves, encompassing low-, medium-, and high-radius configurations with bend angles of 90°, 120°, and 180°. Completion of the test under standard conditions typically spanned approximately ten minutes. To augment environmental realism and heighten drivers’ perceptual awareness of speed, supplementary elements such as roadside vegetation and diverse architectural structures were integrated into the road model. The resultant scene was subsequently exported from RoadRunner R2024a in VTD format and deployed within the driving simulator as the interactive operational environment.

Following scenario construction, participants embodying aggressive, normal, and conservative driving profiles were recruited in a 1:2:1 ratio via targeted questionnaires, thereby ensuring equilibrated sample sizes and enhanced representativeness across archetypes. Throughout the experimental trials, key parameters indicative of driver inputs and vehicular dynamics were meticulously logged, including steering wheel torque, steering wheel angle, vehicle velocity, and vehicle acceleration.

Ultimately, a curated set of ten features was extracted to encapsulate steering habits and overarching driving styles, as shown in Table 1.

### 2.2. Feature Clustering

After obtaining raw feature data, initial screening was performed based on the overall characteristics of the sample dataset, eliminating features exhibiting significant deviations from the dataset. This process yielded 300 validated feature datasets. Subsequent procedures included: normalization via the min-max scaling method, dimensionality reduction through Principal Component Analysis (reducing features from 10D to 3D while retaining > 95% original information), and Fuzzy C-Means clustering for driving style classification. Cluster centroids representing each style were extracted, resulting in sample distributions of aggressive (74 samples), normal (138 samples), and conservative (88 samples) drivers—approximating a 1:2:1 ratio consistent with recruited driver demographics.

(1) $$Y=\left[\begin{array}{ccc} Y_{1} & Y_{2} & Y_{3} \end{array}\right]=Z\left[\begin{array}{ccc} \begin{array}{c} \begin{array}{c} 0.3553\\ 0.3584 \end{array}\\ 0.3273\\ 0.2748 \end{array} & \begin{array}{c} \begin{array}{c} -0.0173\\ -0.0702 \end{array}\\ -0.3028\\ -0.4721 \end{array} & \begin{array}{c} \begin{array}{c} 0.2182\\ -0.0618 \end{array}\\ 0.0953\\ -0.4238 \end{array}\\ \begin{array}{c} 0.2458\\ 0.2089\\ 0.3483 \end{array} & \begin{array}{c} 0.4888\\ 0.6032\\ -0.0793 \end{array} & \begin{array}{c} -0.1849\\ -0.4508\\ -0.0097 \end{array}\\ \begin{array}{c} 0.3445\\ 0.3259\\ 0.3353 \end{array} & \begin{array}{c} -0.1597\\ 0.1828\\ 0.1104 \end{array} & \begin{array}{c} -0.3291\\ 0.5688\\ 0.3008 \end{array} \end{array}\right]$$

(2) $$Z=\left[\begin{array}{ccc} \begin{array}{cc} Z_{1} & Z_{2} \end{array} & \begin{array}{cc} Z_{3} & Z_{4} \end{array} & \begin{array}{cc} \cdots & Z_{10} \end{array} \end{array}\right]$$

where *Z* represents the original ten-dimensional feature data and *Y* denotes the reduced three-dimensional features, dimensionality reduction is achieved by transforming the original data via a projection matrix.

### 2.3. BPNN-Based Recognition and Validation

For driving style identification, this study employs a Backpropagation Neural Network (BPNN). The three-dimensional features derived from clustered sample data serve as inputs, while the membership degrees indicating each sample’s affiliation to the three driving styles (aggressive, normal, conservative) constitute the outputs for neural network training. A single hidden layer is utilized, with its neuron count determined by an empirical formula (detailed below), ultimately fixed at 10 neurons.

(3) $$N=N_{d}\alpha \;\times \left(N_{in}+N_{out}\right)$$

where *N* denotes the desired number of hidden layer neurons, $N_{d}$ represents the total number of training samples, $N_{d}$ represents the total number of training samples, α is a constant ranging from 2 to 10, and $N_{in}$, $N_{out}$ signify the number of neurons in the input layer and output layer, respectively.

**Figure 1:**
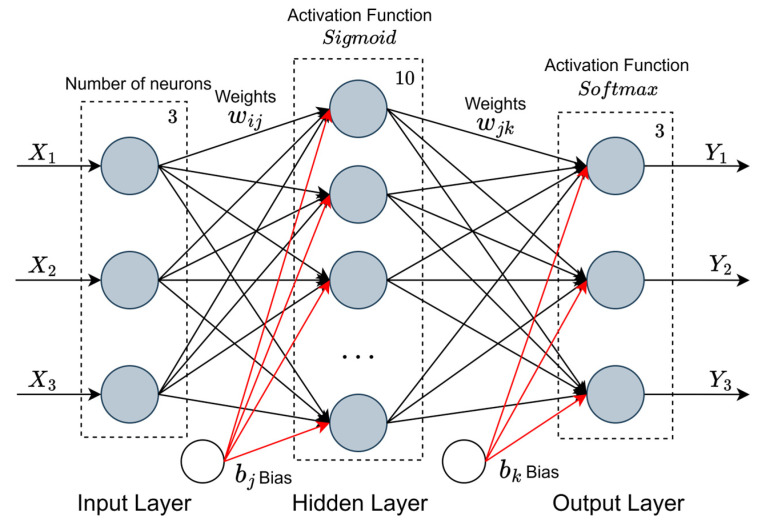
The overall architecture of the neural network.

The activation functions for the hidden layer and output layer are selected as the Sigmoid function and Softmax function, respectively. The overall architecture of the neural network is shown in Figure 1.

**Figure 2:**
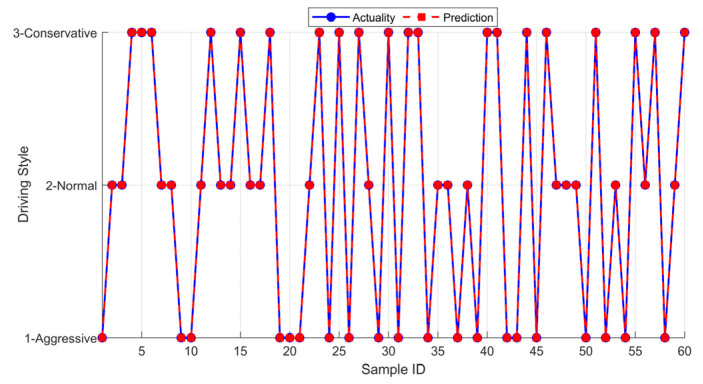
The results of offline driving style recognition.

After the training is completed, the driving style recognition model is obtained; this model accepts reduced three-dimensional features and outputs the probabilities that the features belong to the three driving styles. Here, this study selects the style category with the highest probability as the final identification result of the model. Subsequently, this study randomly selects 20 aggressive-style, 20 normal-style, and 20 conservative-style data samples each from the dimensionality-reduced dataset, inputs them sequentially into the recognition model, compares the actual output with the expected output, where the comparison results are as shown in Figure 2, and the recognition accuracy of this model reaches 100%.

The online recognition process designed in this study is as follows: first, set a flag bit Flag to indicate the completion status of driving style recognition, where Flag = 1 means the current recognition task is completed and no recognition operation is needed; Flag = 0 means the driving style recognition work has not been completed and awaits the recognition process. Since the model trained in this study targets the driving state during the steering process for driving style recognition, the steering wheel angle is monitored to determine the steering state. When its absolute value exceeds the set threshold, it is determined that a steering operation has been performed, and relevant data recording begins until the angle no longer meets the threshold requirement (i.e., the steering operation ends). After recording is completed, the maximum and average values of the corresponding data are obtained according to the data from that period, and a dimensionality reduction operation is performed to yield a three-dimensional feature vector. The feature vector is sent to the pre-trained neural network model, which, having undergone offline training with sample data, identifies the driving style online. The driving style category with the highest probability is selected as the final recognized driving style, and simultaneously, Flag is set to 1, indicating that the driving style recognition task is completed and no further repetitive operation is needed. The online recognition process designed in this study is as follows: first, set a flag bit Flag to indicate the completion status of driving style recognition, where Flag = 1 means the current recognition task is completed and no recognition operation is needed; Flag = 0 means the driving style recognition work has not been completed and awaits the recognition process. Since the model trained in this study targets the driving state during the steering process for driving style recognition, the steering wheel angle is monitored to determine the steering state. When its absolute value exceeds the set threshold, it is determined that a steering operation has been performed, and relevant data recording begins until the angle no longer meets the threshold requirement (i.e., the steering operation ends). After recording is completed, the maximum and average values of the corresponding data are obtained according to the data from that period, and a dimensionality reduction operation is performed to yield a three-dimensional feature vector. The feature vector is sent to the pre-trained neural network model, which, having undergone offline training with sample data, identifies the driving style online. The driving style category with the highest probability is selected as the final recognized driving style, and simultaneously, Flag is set to 1, indicating that the driving style recognition task is completed and no further repetitive operation is needed.

**Figure 3:**
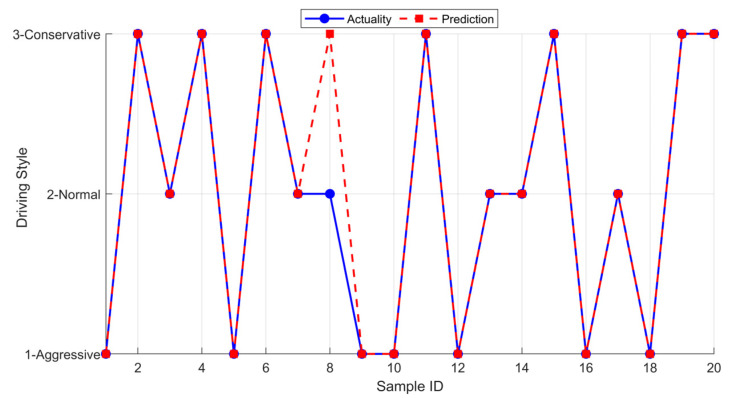
The results of online driving style recognition.

Subsequently, this study arranged 20 drivers recruited from the participants for online identification verification (Figure 3), comparing the model-identified results as predicted output with the clustering division results as actual output, with the outcomes as follows: the identification accuracy rate reached 95%.

## 3. Personalized Closed-Loop Control Strategy for Steering Feel

**Figure 4:**
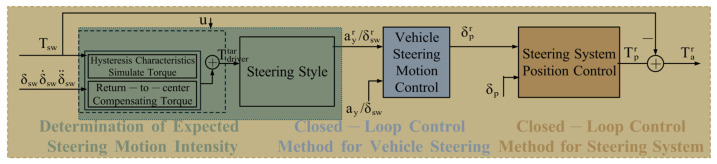
Steering motion quality control architecture.

In the present study, the driver’s anticipated steering motion intensity and the steering system’s positional state are conceptualized as intermediary variables. The proposed control strategy is partitioned into three principal modules: the estimation of the expected steering motion intensity, the regulation of vehicle steering dynamics, and the control of steering system positioning, as illustrated in Figure 4. By prescribing the target steering feel and return-to-center performance metrics, the system implements a closed-loop motion control framework to realize optimal haptic feedback during active steering operations and appropriate return velocity upon release of the steering wheel. This unified integration of steering feel modulation and return-to-center control concomitantly enhances straight-line vehicular stability.

### 3.1. Determination of Expected Steering Motion Intensity

The determination module for the expected steering motion intensity serves to assess the driver’s anticipated steering motion intensity based on their steering input and the current state of the control mechanism. It comprises two parts: determination of the intended torque and steering style (the relationship between the intended torque and steering motion intensity) of the driver. The role of the intended torque determination module of the driver is to determine the torque that accurately represents the intention of the driver according to their steering operation input and the motion state of the control mechanism.

The hysteresis characteristics simulation module controls the hysteresis relationship between the steering torque of the driver and steering motion intensity of the vehicle. In this study, the closed-loop position of the steering system is used to compensate for the hysteresis characteristics of the steering system, while the closed loop of the entire vehicle is used to compensate for the hysteresis characteristics of the chassis dynamics, that is, the double-closed loop compensates for the hysteresis characteristics caused by the electromechanical hydraulic system of the vehicle. Herein, a digital method is used to simulate the hysteresis manually to realize the desired arbitrary hysteresis characteristics. Relevant study has indicated that hysteresis is mainly caused by the inertia, friction, and damping of the system [19]. Therefore, the hysteresis torque simulated in this study includes the friction torque, damping torque, and inertia torque. The friction torque simulation is based on the Fancher model, as expressed in Equation (4), while the damping and inertial torques are based on the linear model, as expressed in Equations (5) and (6), respectively.

(4) $$\left\{\begin{array}{c} \frac{\partial T_{f}}{\partial \delta_{sw}}=\frac{\left|{\dot{\delta }}_{sw}\right|\times \left(T_{env}-T_{f}\right)}{\beta (\delta_{sw},v)}\\ T_{env}=-sign\left({\dot{\delta }}_{sw}\right)T_{f}(\delta_{sw},v) \end{array}\right.$$

(5) $$T_{d}=-K_{d}\left(\delta_{sw},v\right){\dot{\delta }}_{sw}$$

(6) $$T_{i}=-K_{i}\left(\delta_{sw},v\right){\ddot{\delta }}_{sw}$$

where $T_{f}$, $T_{d}$, and $T_{i}$ represent the simulated friction, damping, and inertia torques, respectively; $T_{env}$ and *β* the set friction torque and friction relaxation angle, respectively, both of which vary with respect to the speed and steering wheel angle. $K_{d}$ and $K_{i}$ represent the damping coefficient and inertia coefficient, respectively, both of which vary with respect to the speed and angle.

**Figure 5:**
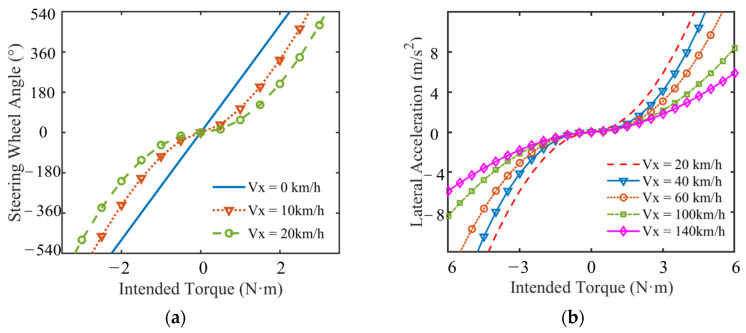
Relationship between the intended torque and the steering motion intensity of a vehicle. (**a**) Low Speed. (**b**) High Speed.

The principal function of the return-to-center compensation torque is to regulate the steering wheel’s reversion to its neutral position at an adaptive velocity, applicable either subsequent to the driver’s release of the steering wheel or during sustained holding thereof. This compensatory torque is activated exclusively under conditions wherein the driver releases or maintains grip on the steering wheel; conversely, it is deactivated during active steering maneuvers or when the driver commences the return-to-center action. Under these latter scenarios, the compensation torque is nullified to zero. As illustrated in Figure 4, the computation of the return-to-center compensation torque can be bifurcated into two sequential stages. Foremost, the target steering wheel return-to-center velocity is determined predicated on the driver’s steering inputs and the contemporaneous kinematic state of the control mechanism. from which the desired steering wheel position is established. Subsequently, the return-to-center compensation torque is calculated, aiming to track this specified position. The steering style module sets the relationship between the intended torque and steering motion intensity of the driver. The corresponding relationship between the intended torque of the driver and vehicle steering motion intensity is obtained, as shown in Figure 5.

### 3.2. Closed-Loop Control Method for Vehicle Steering

**Figure 6:**
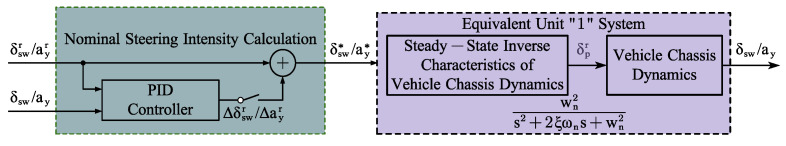
Closed-loop control method for vehicle steering motion.

The vehicle steering motion closed-loop control module is engineered to compute the requisite positional state of the steering system essential for realizing the targeted steering motion intensity. Premised on the principles of internal model control and closed-loop regulation, this module encompasses two primary components: the inverse steady-state characteristics of the vehicle chassis dynamics and the nominal steering motion intensity computation submodule, as depicted in Figure 6.

The nominal steering motion intensity computation module ascertains the nominal steering motion intensity by integrating the actual and reference steering intensities, thereby enabling the derivation of a corresponding steering system positional control variable that actualizes the reference steering motion intensity. Furthermore, this module mitigates calibration discrepancies within the steady-state inverse characteristics of the vehicle chassis dynamics, thereby diminishing the requisite precision for inverse characteristic calibration. Concurrently, it modulates the equivalent closed-loop response bandwidth of the vehicle chassis dynamics to impart predefined dynamic attributes. To this end, the module employs a proportional–integral–derivative (PID) control architecture augmented with a direct feedthrough component. The steady-state inverse characteristics submodule of the vehicle chassis dynamics serves to compute the appropriate steering system positional control variable predicated on the nominal steering motion intensity. Additionally, it rectifies the steady-state nonlinearities inherent to the vehicle chassis dynamics while normalizing the gain of the controlled chassis subsystem.

### 3.3. Closed-Loop Control Method for Steering System

**Figure 7:**
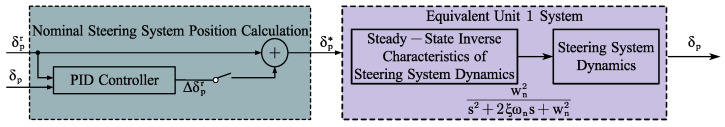
Closed-loop control method for steering system position.

The steering system position closed-loop control module is engineered to compute the assistive steering torque essential for realizing the target positional state of the steering system. Premised on the principles of internal model control and closed-loop regulation, this module encompasses two primary components: the inverse steady-state characteristics of the steering system dynamics and the nominal steering system position computation submodule. The nominal steering system position computation module ascertains the nominal steering system position by integrating the actual and reference positional states, thereby generating a corresponding equivalent active steering torque that facilitates the realization of the reference steering system position. The module’s architecture is illustrated in Figure 7 and incorporates a proportional–integral–derivative (PID) control framework augmented with a direct feedthrough element. The parameters of the PID controller were tuned based on the frequency domain response analysis of the steering system. Given the controlled system and the desired transfer function of the closed-loop system, the PID control parameters are presented in Table 2. As observed from the table, the integral coefficients at different vehicle speeds are all zero. This is attributed to the fact that both the controlled system and the desired system have a gain of “1”, resulting in no steady-state deviation. However, to mitigate steady-state errors induced by variations in the dynamic characteristics of the steering system, $K_{i}$ is typically set to an appropriate non-zero value.

## 4. Verification of Adaptability of Steering Feel

In this study, the experiments on different speeds and different road surfaces were simulated using the driving simulator.

**Figure 8:**
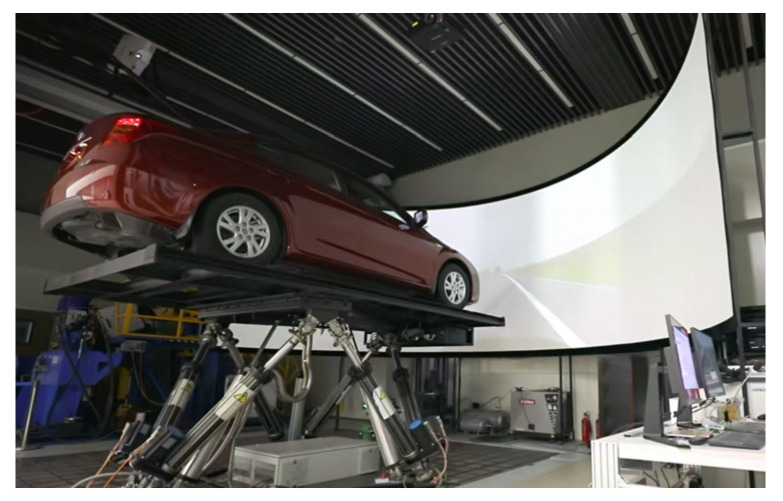
ASCL driving simulator.

The driver-in-the-loop simulation platform, as shown in Figure 8, is developed using dSPACE and a driving simulator and comprises six main components: the driving environment simulation system, vehicle dynamics, micro-autobox, dSPACE-simulator, motion platform, and driving cockpit. Their functions are as follows: (1) the vehicle dynamics component calculates the response of the vehicle to driver inputs and the electronic control system, thereby relaying these responses to the motion platform, the parameters of the vehicle model is shown in Table 3; (2) the driving environment simulation system, using VTD (Virtual Test Drive) software, simulates the driving conditions such as the roads, traffic, and weather; (3) the dSPACE-simulator simulates the CAN bus communication of the vehicle; (4) the micro-autobox runs the electronic control strategies under test; (5) the motion platform provides the driver with a sense of vehicle movement; (6) the driving cockpit collects the input operations of the driver, including the steering wheel, accelerator pedal, brake pedal, and gear position. It also provides real-time force feedback and displays information such as instrument readings and turn signals.

**Figure 9:**
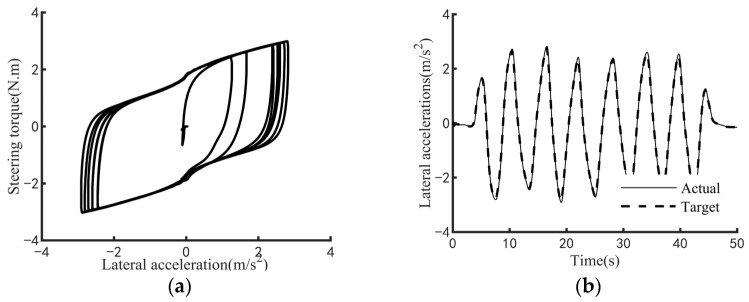
Test results for normal driving style. (**a**) Diagram of steering feel. (**b**) Diagram of lateral acceleration tracking.

**Figure 10:**
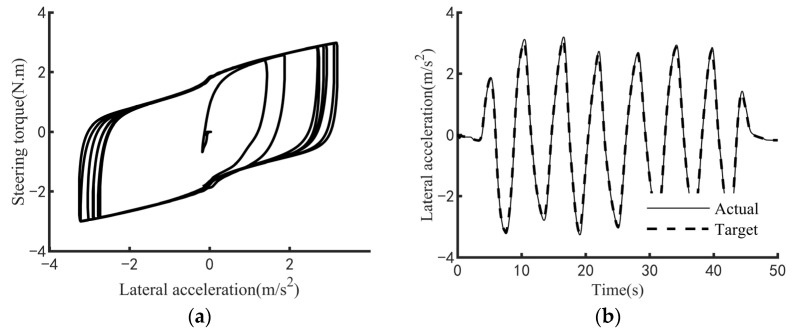
Test results for aggressive driving style. (**a**) Diagram of steering feel. (**b**) Diagram of lateral acceleration tracking.

**Figure 11:**
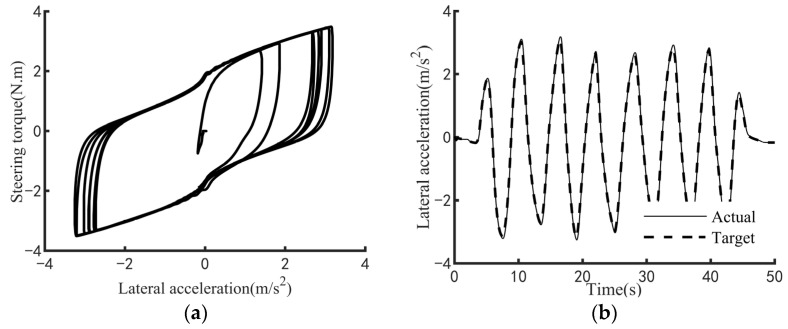
Test results for conservative driving style. (**a**) Diagram of steering feel. (**b**) Diagram of lateral acceleration tracking.

**Figure 12:**
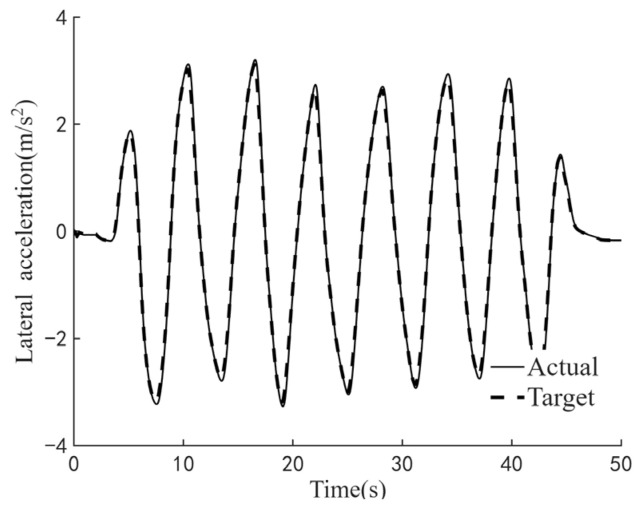
Test results for aggressive driving style with MPC controller.

The efficacy of the steering feel control strategy pertains to its capacity to elicit the targeted steering response while regulating the interplay between the driver’s applied steering torque and the resultant steering motion intensity. To comprehensively evaluate this efficacy across a broad spectrum of vehicular velocities and steering amplitudes, three standardized maneuverability and stability assessment protocols are employed: the static steering test, the double-lane-change test, and the on-center steering test. Specifically, the static steering test appraises steering feel under stationary conditions or at ultra-low speeds; the double-lane-change test examines performance at low speeds; and the on-center steering test assesses efficacy at medium-to-high speeds. Figure 9, Figure 10 and Figure 11 respectively demonstrate the effectiveness of the control strategy proposed in this paper on steering feel and vehicle motion control under normal driving style, aggressive driving style and conservative driving style. The test results demonstrate that the proposed steering feel control method successfully achieves the desired steering feel across a comprehensive range of speeds and turning angles, thereby ensuring a precise correlation between the driver’s steering torque and the intensity of steering motion. Figure 12 shows the control effect achieved by using the MPC controller. The control strategy using MPC shows no significant difference in control effect from the method proposed in this paper, while the PID controller has the advantage of higher computational efficiency.

## 5. Conclusions

In response to the growing demand for personalized steering experiences in modern vehicles, this study proposes a comprehensive control strategy that integrates driving style recognition with a closed-loop steering feel control framework. The key contributions and findings are summarized as follows:

(1) Driving Style Recognition: A robust recognition model based on a Backpropagation Neural Network (BPNN) was developed, utilizing dimensionality-reduced feature data extracted from driver behavior. The model achieved high accuracy in both offline (100%) and online (95%) validations, enabling real-time identification of aggressive, normal, and conservative driving styles.
(2) Personalized Steering Feel Control: A three-module closed-loop control architecture was designed, incorporating the estimation of expected steering motion intensity, vehicle steering dynamics control, and steering system positioning control. This structure allows for dynamic adaptation of steering feel according to the identified driving style and real-time vehicle states.
(3) Experimental Validation: Through driver-in-the-loop simulations under various speed and road conditions, the proposed strategy demonstrated effective and consistent performance in delivering the target steering feel. Standard tests—including static steering, double-lane change, and on-center steering—confirmed that the system successfully maintains desired steering torque–motion relationships across different operational scenarios.
(4) Integration of Steering Feel and Return-to-Center Control: By incorporating hysteresis compensation and adaptive return-to-center logic, the system not only enhances steering feedback during active maneuvers but also improves straight-line stability and steering recentering behavior.

In conclusion, this study presents a feasible and effective approach to achieving personalized steering feel through intelligent style recognition and adaptive closed-loop control. Future work will focus on further refining the recognition model under more diverse driving conditions and extending the control strategy to steer-by-wire systems. Furthermore, to address the limitation of the current one-time recognition logic (where the Flag bit locks the style), we plan to develop a dynamic updating mechanism. This will allow the system to continuously monitor and adapt to changes in driver behavior or driving style during long-distance driving. Additionally, transferring this strategy from a simulator to a real vehicle presents practical challenges, such as signal noise from onboard sensors and the computational constraints of automotive Electronic Control Units (ECUs). Addressing these issues through robust filtering algorithms and code optimization will be a critical focus of our upcoming on-road testing phases.

## Figures and Tables

**Table 1:** Features representing the driving style and steering habits of drivers.

Features Representing the Driving Style and Steering Habits of Drivers	
Average steering wheel angle multiplied by vehicle speed	Average steering wheel torque
Maximum steering wheel angle multiplied by vehicle speed	Peak steering wheel torque
Average steering wheel angular velocity	Peak steering wheel angular velocity
Average lateral acceleration	Peak lateral acceleration
Average yaw rate	Peak yaw rate

**Table 2:** PID control parameters of the steering system at different vehicle speeds.

Parameter	0	20	40	60	80	100	120	140
P	9.10	8.52	8.21	6.37	4.23	2.78	2.02	1.69
I	0	0	0	0	0	0	0	0
D	0.12	0.23	0.56	0.68	0.87	0.86	0.84	0.73

**Table 3:** Key parameters of the vehicle model.

Parameter	Value	Unit
Vehicle Mass	1800	kg
Distance from CG to Rear Axle	1.02	m
Distance from CG to Front Axle	1.68	m
Steering Gear Ratio	16	-
Tire Cornering Stiffness (Front)	68,000	N/rad
Tire Cornering Stiffness (Rear)	64,000	N/rad
Yaw Moment of Inertia	1536.7	kg·m^2^

## Data Availability

The datasets generated during and/or analyzed during the current study are not publicly available due to privacy and ethical restriction.

## Abbreviations

The following abbreviations are used in this manuscript:

EPS	Electric power steering
PID	Proportional–integral–derivative
VTD	Virtual Test Drive
